# Supplementary material for: A Systematic Review of Heat Load in Australian Livestock Transported by Sea
Source: Animals (Basel). 2018 Sep 27;8(10):164. doi: 10.3390/ani8100164 (PMC6210166; doi:10.3390/ani8100164)
Supplement: Supplementary file 1 [file animals-08-00164-s001.pdf]

SUPPLEMENTARY MATERIAL

Additional Supplementary Material can be found in the online version of this article.

Appendix S1.

Protocol for identifying literature relevant to heat load and sea transport of Australian livestock.

Appendix S2.

Figure S1. PRSIMA flow diagram [1] for inclusion of the 92 publications identified in our literature search.

Figure S2. Year of publication for the 93 publications identified in our literature search. Five-year intervals were used, except for 2015−2018, which was four years.

Table S1. PRISMA checklist.

Table S2. Classification and appraisal of each literature item.

SUPPLEMENTARY MATERIAL

Protocol for identifying literature relevant to heat load and sea transport of Australian livestock.

To minimise the risk of reporting bias due to incomplete retrieval of research, we followed the PRISMA guidelines for systematic reviews [1]. We used an electronic database (Google Scholar), the Murdoch University library catalogue, and our professional networks to identify relevant journal articles, books, unpublished reports, conference proceedings and theses. We conducted searches of literature published in English. We conducted a series of searches in all years for each of the common (sheep, cattle) and the scientific names (*Ovis aries*, *Bos taurus*/*indicus*) of the species of interest and the terms 'heat', 'heat load' and 'heat stress'. We then combined these iteratively with the following search terms: 'Australia', 'live export' and 'sea transport' to ensure that no relevant studies were missed. For unpublished studies, we searched the databases of the funding agency commissioning research into live export in Australia: Meat and Livestock Australia. We also searched the websites of the two most prominent animal welfare advocacy groups involved: RSPCA Australia and Animals Australia. We also searched the bibliographies of the literature obtained and cited some non-Australian studies of relevance that were cited by Australian studies that we found.

We found 93 literature items in total, comprising published and unpublished items (Figure S1). One item [2] was excluded from the study due to the unavailability of a full-text. We displayed literature found by year and publication type. Most literature items found were contemporary, with 79% published since 2000 (Figure S2). We did not extract effect and precision estimates (as is desirable when conducting a systematic review focused on a

specific question) [1] because our review topic was so broad and involved qualitative information. To summarize the findings and appraise the quality of the evidence presented in the literature, we tabulated study characteristics, quality and outcomes (Tables S2). Specifically, each piece of literature was classified by risk of bias as influenced by publication type and study type, and ranked as low (presents original data and peer-reviewed), medium (presents original data but not peer-reviewed *or* does not present original data but is peer-reviewed) or high (does not present original data and is not peer-reviewed).

References

1. Moher, D.; Liberati, A.; Tetzlaff, J.; Altman, D.G.; Group, P. Preferred reporting items for systematic reviews and meta-analyses: The prisma statement. *PLoS Medicine* **2009**, *6*, e1000097.
2. Hedlefs, R. *Factors influencing mortality and wastage of slaughter cattle transported from Queensland to Japan by sea*; Queensland Department of Primary Industries: Brisbane, Australia, 1988.

Figure S1. PRSIMA flow diagram [1] for inclusion of the 93 publications identified in our literature search.

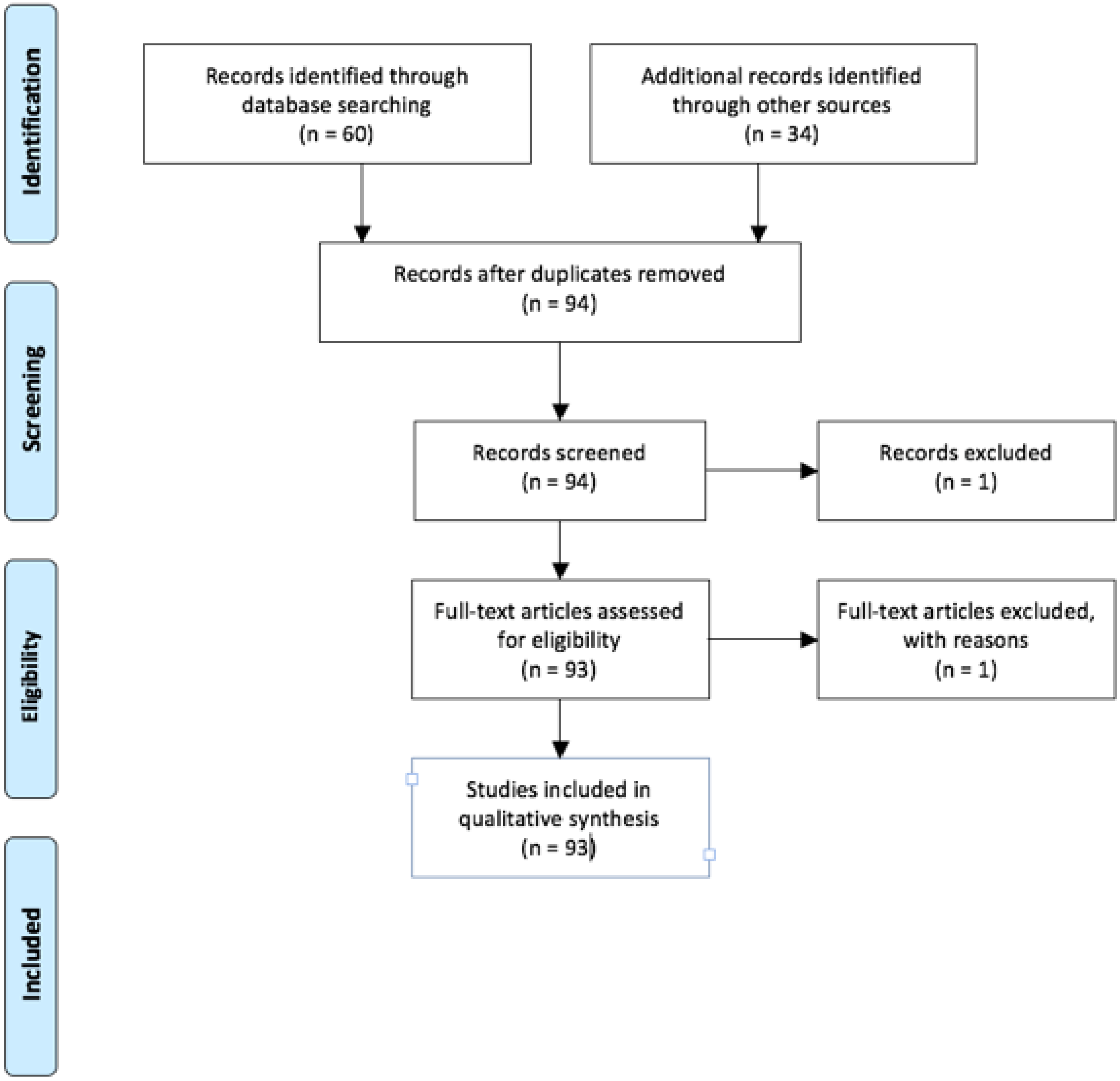

Figure S2. Year of publication for the 93 publications identified in our literature search. Five-year intervals were used, except for 2015−2018, which was four years.

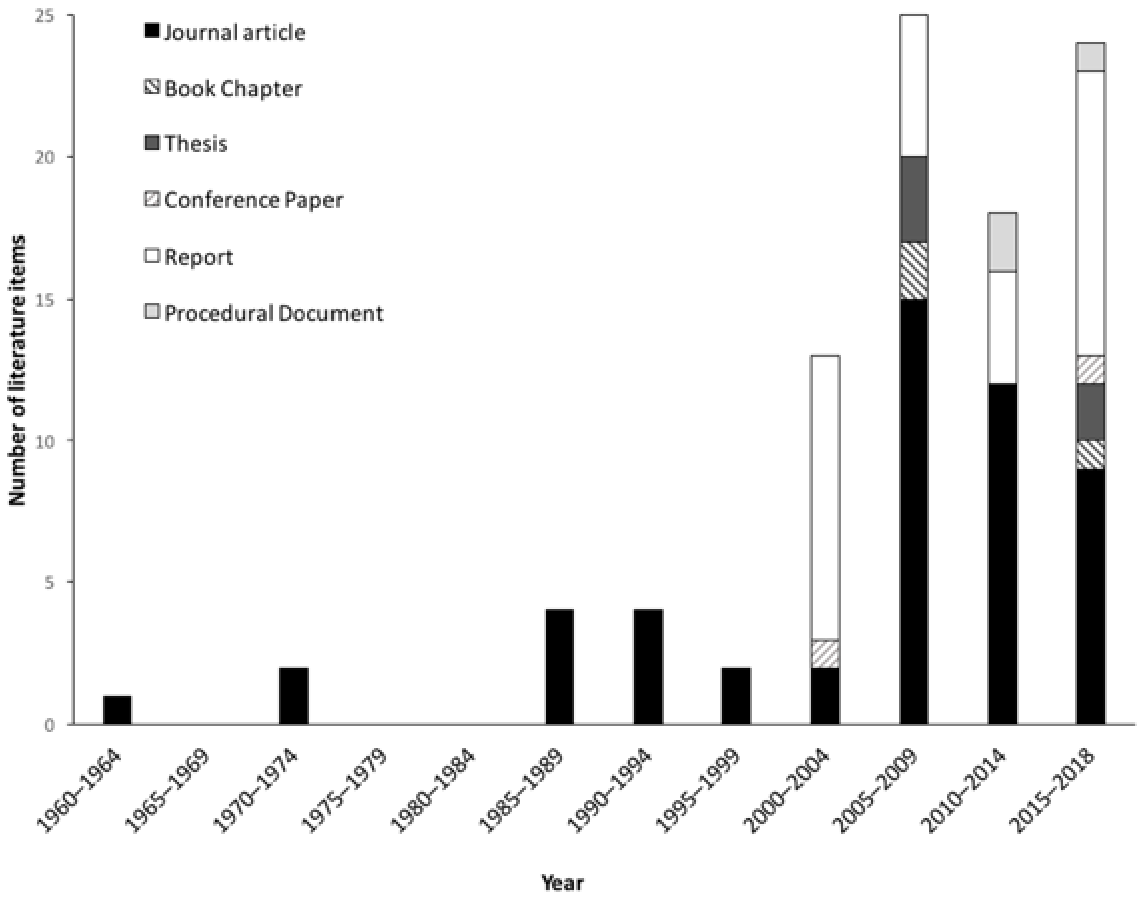

Table S1. PRISMA checklist [1].

Section/topic	#	Checklist item	Reported on page #
**TITLE**			
Title	1	Identify the report as a systematic review, meta-analysis, or both.	1
**ABSTRACT**			
Structured summary	2	Provide a structured summary including, as applicable: background; objectives; data sources; study eligibility criteria, participants, and interventions; study appraisal and synthesis methods; results; limitations; conclusions and implications of key findings; systematic review registration number.	1
**INTRODUCTION**			
Rationale	3	Describe the rationale for the review in the context of what is already known.	3
Objectives	4	Provide an explicit statement of questions being addressed with reference to participants, interventions, comparisons, outcomes, and study design (PICOS).	3
**METHODS**			
Protocol and registration	5	Indicate if a review protocol exists, if and where it can be accessed (e.g., Web address), and, if available, provide registration information including registration number.	Supporting Material
Eligibility criteria	6	Specify study characteristics (e.g., PICOS, length of follow-up) and report characteristics (e.g., years considered, language, publication status) used as criteria for eligibility, giving rationale.	Supporting Material
Information sources	7	Describe all information sources (e.g., databases with dates of coverage, contact with study authors to identify additional studies) in the search and date last searched.	Supporting Material
Search	8	Present full electronic search strategy for at least one database, including any limits used, such that it could be repeated.	Supporting Material
Study selection	9	State the process for selecting studies (i.e., screening, eligibility, included in systematic review, and, if applicable, included in the meta-analysis).	Supporting Material
Data collection process	10	Describe method of data extraction from reports (e.g., piloted forms, independently, in duplicate) and any processes for obtaining and confirming data from investigators.	Supporting Material
Data items	11	List and define all variables for which data were sought (e.g., PICOS, funding sources) and any assumptions and simplifications made.	Supporting Material
Risk of bias in individual studies	12	Describe methods used for assessing risk of bias of individual studies (including specification of whether this was done at the study or outcome level), and how this information is to be used in any data synthesis.	Supporting Material
Summary measures	13	State the principal summary measures (e.g., risk ratio, difference in means).	NA
Synthesis of results	14	Describe the methods of handling data and combining results of studies, if done, including measures of consistency (e.g., I^2) for each meta-analysis.	NA

Table S2. Classification and appraisal of each literature item.

**Study**	**Year**	**Publication type**	**Livestock species**		**Study type**					**Original data (Y/N)**	**Risk of bias**
			**Sheep**	**Cattle**	**Experiment**	**Monitoring and epidemiology**	**Review**	**Modelling**	**Procedural document**		
Fisher and Jones	2008	Book chapter	✓	✓			✓			N	High
Phillips	2008	Book chapter	✓	✓			✓			N	High
Zhang and Phillips	2018	Book chapter	✓	✓			✓			N	High
More et al.	2003	Conference proceedings	✓	✓		✓				Y	Moderate
Collins et al.	2016	Conference proceedings	✓		✓					Y	Moderate
Wodzicka	1960	Peer-reviewed article	✓		✓					Y	Low
Johnson	1970	Peer-reviewed article		✓	✓					Y	Low
Maskrey	1974	Peer-reviewed article	✓		✓					Y	Low
Norris et al.	1989a	Peer-reviewed article	✓			✓				Y	Low
Norris et al.	1989b	Peer-reviewed article	✓			✓				Y	Low
Norris et al.	1989c	Peer-reviewed article	✓			✓				Y	Low
Richards et al.	1989	Peer-reviewed article	✓			✓				Y	Low
Higgs et al.	1991	Peer-reviewed article	✓			✓				Y	Low
Richards et al.	1991	Peer-reviewed article	✓			✓				Y	Low
Higgs et al.	1993	Peer-reviewed article	✓			✓				Y	Low
Blackshaw and Blackshaw	1994	Peer-reviewed article		✓			✓			N	Moderate
Dixon et al.	1999	Peer-reviewed article	✓		✓					Y	Low
Higgs et al.	1999	Peer-reviewed article	✓			✓				Y	Low

Table S2 continued.

Study	Year	Publication type	Livestock species		Study type					Original data (Y/N)	Risk of bias
			Sheep	Cattle	Experiment	Monitoring and epidemiology	Review	Modelling	Procedural document		
Norris et al.	2003	Peer-reviewed article		✓		✓				Y	Low
Srikandakumar et al.	2003	Peer-reviewed article	✓		✓					Y	Low
Bortolussi et al.	2005	Peer-reviewed article		✓			✓			N	Moderate
Norris	2005	Peer-reviewed article	✓	✓			✓			N	Moderate
Petherick	2005	Peer-reviewed article		✓			✓			N	Moderate
Beatty et al.	2006	Peer-reviewed article		✓	✓					Y	Low
Beatty et al.	2007	Peer-reviewed article		✓	✓					Y	Low
Petherick	2007	Peer-reviewed article	✓	✓			✓			N	Moderate
Adams and Thornber	2008	Peer-reviewed article	✓	✓			✓			N	Moderate
Gaughan et al.	2008	Peer-reviewed article		✓		✓				Y	Low
Beatty et al.	2008a	Peer-reviewed article	✓		✓					Y	Low
Beatty et al.	2008b	Peer-reviewed article		✓	✓					Y	Low
Savage et al.	2008	Peer-reviewed article	✓		✓					Y	Low
Stinson	2008	Peer-reviewed article	✓	✓			✓			N	Moderate
Gaughan et al.	2009	Peer-reviewed article		✓	✓					Y	Low
Petherick and Phillips	2009	Peer-reviewed article	✓	✓				✓		N	Moderate
Gaughan et al.	2010	Peer-reviewed article		✓	✓					Y	Low

Table S2 continued.

Study	Year	Publication type	Livestock species		Study type					Original data (Y/N)	Risk of bias
			Sheep	Cattle	Experiment	Monitoring and epidemiology	Review	Modelling	Procedural document		
Stockman et al.	2011	Peer-reviewed article	✓		✓					Y	Low
Alhidary et al.	2012a	Peer-reviewed article	✓		✓					Y	Low
Alhidary et al.	2012b	Peer-reviewed article	✓		✓					Y	Low
Gaughan et al.	2013	Peer-reviewed article		✓	✓					Y	Low
Pines and Phillips	2013	Peer-reviewed article	✓			✓				Y	Low
Schipp	2013	Peer-reviewed article	✓	✓			✓			N	High
Caulfield et al.	2014	Peer-reviewed article	✓	✓			✓			N	Moderate
Chauhan et al.	2014a	Peer-reviewed article	✓		✓					Y	Low
Chauhan et al.	2014b	Peer-reviewed article	✓		✓					Y	Low
Foster and Overall	2014	Peer-reviewed article	✓	✓			✓			N	Moderate
Moore et al.	2014	Peer-reviewed article		✓		✓				Y	Low
Alhidary et al.	2015	Peer-reviewed article	✓		✓					Y	Low
Chauhan et al.	2015	Peer-reviewed article	✓		✓					Y	Low
DiGiacomo et al.	2016	Peer-reviewed article	✓		✓					Y	Low
Phillips	2016	Peer-reviewed article	✓				✓			N	Moderate
Zhang et al.	2017	Peer-reviewed article	✓			✓				Y	Low
Jackson and Adamson	2018	Peer-reviewed article	✓				✓			N	Moderate

Table S2 continued.

Study	Year	Publication type	Livestock species		Study type					Original data (Y/N)	Risk of bias
			Sheep	Cattle	Experiment	Monitoring and epidemiology	Review	Modelling	Procedural document		
Santurtun and Phillips	2018	Peer-reviewed article	✓		✓					Y	Low
Sinclair et al.	2018	Peer-reviewed article	✓	✓		✓				Y	Low
Zhang et al.	2018	Peer-reviewed article	✓		✓					Y	Low
Commonwealth of Australia	2011	Procedural document	✓	✓					✓	N	High
Department of Agriculture, Fisheries and Forestry	2013	Procedural document	✓	✓					✓	N	High
Department of Agriculture and Water Resources	2018	Procedural document	✓	✓					✓	N	High
MAMIC Pty Ltd	2000	Report	✓	✓		✓				Y	Moderate
Sparke et al.	2001	Report		✓			✓			N	High
MAMIC Pty Ltd	2002	Report	✓	✓		✓				Y	Moderate
Gaughan et al.	2003	Report		✓		✓				Y	Moderate
Keniry	2003	Report	✓	✓			✓			N	Hgh
Maunsell Australia	2003	Report	✓	✓				✓		N	High
Tudor et al.	2003	Report		✓	✓					Y	Moderate
Barnes et al.	2004	Report	✓	✓			✓			N	High
Maunsell Australia	2004	Report	✓			✓				Y	Moderate
McCarthy	2005	Report	✓	✓		✓				Y	Moderate
Byrne et al.	2006	Report	✓	✓		✓				Y	Moderate

Table S2 continued.

Study	Year	Publication type	Livestock species		Study type					Original data (Y/N)	Risk of bias
			Sheep	Cattle	Experiment	Monitoring and epidemiology	Review	Modelling	Procedural document		
Barnes et al.	2008	Report	✓	✓	✓					Y	Moderate
Ferguson et al.	2008	Report	✓	✓			✓			N	High
Kennedy	2008	Report		✓	✓					Y	Moderate
Banney et al.	2009	Report	✓	✓			✓			N	High
Farmer	2011	Report	✓	✓			✓			N	High
Ferguson and Lea	2013	Report	✓		✓					Y	Moderate
Shiell et al.	2013	Report	✓				✓			N	High
Gobbett et al.	2014	Report	✓	✓			✓			N	High
Perkins et al.	2015	Report		✓		✓				Y	Moderate
McCarthy and Banhazi	2016	Report	✓	✓			✓			N	High
McCarthy and Fitzmaurice	2016	Report	✓	✓			✓			N	High
Norman	2016	Report	✓	✓		✓				Y	Moderate
Norman	2017	Report	✓	✓		✓				Y	Moderate
Wickham et al.	2017	Report	✓	✓			✓			N	High
Wiebe et al.	2017	Report	✓	✓				✓		N	High
Animals Australia	2018	Report	✓	✓			✓			N	High
Australian Veterinary Association	2018	Report	✓				✓			N	High
McCarthy	2018	Report	✓				✓			N	High
Beatty	2005	Thesis		✓	✓					Y	Moderate
Outschoorn	2005	Thesis				✓				Y	Moderate
Stockman	2006	Thesis	✓		✓					Y	Moderate

Table S2 continued.

Study	Year	Publication type	Livestock species		Study type					Original data (Y/N)	Risk of bias
			Sheep	Cattle	Experiment	Monitoring and epidemiology	Review	Modelling	Procedural document		
Aguilar Gainza	2015	Thesis	✓		✓					Y	Moderate
Tait	2015	Thesis		✓	✓					Y	Moderate
